# Heavy Metal Contamination and Risk Assessment in Soil–Wheat/Corn Systems near Metal Mining Areas in Northwestern China

**DOI:** 10.3390/biology14111475

**Published:** 2025-10-23

**Authors:** Shenghui Xu, Mingyang Yun, Yan Wang, Kaiwang Liu, Ao Wu, Shuning Li, Yanfang Su, Shengli Wang, Hongmei Kang

**Affiliations:** 1 School of Civil and Hydraulic Engineering, Lanzhou University of Technology, Langongping Road 287, Lanzhou 730050, China; xush@lut.edu.cn (S.X.); yun15937813190@163.com (M.Y.); ywang0209@lut.edu.cn (Y.W.); gslzlkw@gmail.com (K.L.); wuao555@163.com (A.W.); lishuning0508@foxmail.com (S.L.); 2 Key Laboratory of Industrial Waste(s) Recycling and Regulation, Gansu Academy of Eco-Environmental Sciences, Lanzhou 730030, China; 13893481671@163.com; 3 Gansu Key Laboratory for Environmental Pollution Prediction and Control, College of Earth and Environmental Sciences, Lanzhou University, Tianshui South Road 222, Lanzhou 730000, China; 4 Institute of Biology, Gansu Academy of Sciences, Lanzhou 730000, China; lzkanghm@163.com

**Keywords:** farmland soils, heavy metal contamination, spatial distribution, risk assessment, urban oasis

## Abstract

**Simple Summary:**

With the accelerating pace of modernization and industrialization, the rapid development of mining and metallurgical sectors has led to farmland soil contamination, consequently jeopardizing local ecosystems, food security, and residents’ health. Heavy metals are known to pose risks to human health through the food chain. To comprehensively assess the potential hazards within the soil–crop system near smelting areas in Jinchang City, China, this study conducted multi-point field investigations and analyses of heavy metal distribution in farmland soils and crops around smelting facilities in this mining city. The research reveals the spatial distribution characteristics of Ni (nickel), Cu (copper), and Co (cobalt) in local soils, wheat, and corn, along with associated human health risks, while identifying key contaminated zones. These findings provide valuable reference for subsequent studies on the soil–crop system in the investigated area and establish fundamental data for remediation of heavy metal contamination in local farmland soils.

**Abstract:**

Heavy metals in farmland soils pose severe threats to agricultural productivity and food safety. To investigate contamination in the soil–wheat/corn system, 24 sets of adjacent farmland soil, wheat, and corn plant samples were collected near metal smelting facilities in Jinchang City, a typical urban oasis in northwestern China. Concentrations of Ni (nickel), Cu (copper), and Co (cobalt) were measured. Results indicated mean soil concentrations of 143.66 mg kg^−1^ (Ni), 130.00 mg kg^−1^ (Cu), and 24.04 mg kg^−1^ (Co), all exceeding background values for Gansu Province, confirming that the sampling sites exhibit varying degrees of contamination with Ni, Cu, and Co. Correlation analyses revealed strong intermetal relationships (Ni, Cu, Co; *p* < 0.01), while spatial distribution patterns showed that Ni in wheat and corn grains closely mirrored soil Ni distribution. The bio-concentration factor (BCF) for wheat roots surpassed that of corn roots, highlighting wheat’s greater susceptibility to heavy metal uptake. Heavy metal levels in crop organs exceeded limits set by the Safety Guidelines for Feed Additives. Geo-accumulation indices and potential ecological risk assessments demonstrated substantial metal accumulation and varying ecological risks, with contamination levels ranked as Cu > Ni > Co. Non-carcinogenic hazard indices indicated elevated health risks for children consuming locally grown wheat and corn. This study provides a scientific foundation for crop rotation strategies and soil remediation in the region.

## 1. Introduction

The extraction, beneficiation, and smelting of non-ferrous metal deposits have driven industrial growth and economic development, yet simultaneously inflicted severe environmental pollution [1,2]. Rapid industrialization has led to soil heavy metal concentrations far exceeding regulatory thresholds, raising public concern over soil environmental safety [3,4,5]. Heavy metals from smelting slag and tailings infiltrate adjacent soils via surface runoff during rainfall, persisting indefinitely due to their non-biodegradable nature. Such pollution not only alters soil microbial community structure and function but may also contaminate groundwater through vertical permeation [6,7]. From an agricultural perspective, heavy metal pollution reduces crop yields or induces total harvest failure. Studies demonstrated that soil copper (Cu) contamination reduced wheat and corn germination rates by 10% and 16%, respectively, with wheat root length stunted to 0.03 cm and corn root growth rates declining 12-fold under Cu stress [8]. Human health risks are equally critical: excessive heavy metal intake triggers acute or chronic toxicity [9,10], causing irreversible damage to immune, reproductive, and nervous systems [11,12]. Notably, heavy metals enter humans via food chain accumulation or inhalation, with toxic element uptake in vegetables and crops linked not only to soil contamination but also to exogenous inputs such as atmospheric deposition, transportation emissions, and sewage sludge application [13]. For instance, long-term wastewater irrigation has led to the accumulation of Ni, Cd, and Cr in wheat grains in a suburban agricultural town in eastern Pakistan [14]. Wang et al. [15] identified atmospheric deposition and transportation as the dominant sources of heavy metals in the western Hexi Corridor. We employed the term "heavy metal" in this study. While we acknowledge the ongoing debate regarding its precise definition, the term has been retained to maintain consistency and ensure direct comparability with previous research.

Areas surrounding metal smelters and mines, subjected to prolonged heavy metal dust deposition and wastewater infiltration, represent critical hotspots for soil heavy metal accumulation. In Jiyuan City, Henan Province, heavy metal contamination in agricultural soils surrounding the Yuguang Smelter has been directly linked to prolonged metallurgical activities. This persistent pollution has resulted in non-carcinogenic health risks for all local children, constituting a significant health threat to residents [16]. Further investigations in Hunan Province revealed that manganese (Mn), cadmium (Cd), lead (Pb), copper (Cu), zinc (Zn), and chromium (Cr) levels in soils near manganese mines surpassed background values by factors of 16.3, 15.4, 15.0, 9.9, 6.1, and 1.1, respectively. Among these, Mn, Cd, and Pb exhibited severely contaminated levels, correlating with elevated potential ecological risks [17]. Notably, studies by Xiang et al. [3] in the Yangtze River Delta highlighted that cereals exhibited markedly stronger heavy metal bioaccumulation than vegetables or fruits, while children exhibit significantly greater health sensitivity to heavy metal exposure than adults. In response, the World Health Organization (W.H.O.) and national regulatory bodies have rigorously revised maximum permissible heavy metal concentrations in food products. To quantify non-carcinogenic health risks in mining and smelting zones, researchers widely employ hazard quotient (*HQ*) and hazard index (*HI*) models established by the US Environmental Protection Agency (USEPA), underscoring their global applicability in health risk assessment frameworks [15,18,19,20].

According to Wang et al. [15], metal processing and smelting activities are the primary sources of heavy metal contamination in agricultural soils across the Hexi Corridor, with the highest concentrations observed in central regions. Mining areas represent the most heavily contaminated land-use type [20]. Jinchang City, a major production base for nickel (Ni), copper (Cu), and cobalt (Co) in China and renowned as the “Nickel Capital”, has experienced significant environmental degradation due to prolonged resource extraction [19,21]. Emissions from heavy metal-laden exhaust gases and dust from tailings ponds have driven the accumulation of toxic elements in farmland soils, posing potential risks to crops [17]. Wheat and corn, as staple crops in Jinchang, are critical subjects for investigating heavy metal pollution characteristics and conducting health risk assessments in peri-mining areas. Such research not only enhances public and scientific understanding of regional contamination but also provides actionable insights for mitigating heavy metal exposure and advancing environmental conservation, underscoring its practical significance.

This study aimed to (1) determine the concentrations and spatial distribution patterns of Ni, Cu, and Co in soils and staple crops (wheat and corn), and (2) evaluate and compare the degree of soil pollution and associated health risks posed by wheat and corn consumption.

## 2. Materials and Methods

### 2.1. Study Area

**Figure 1:**
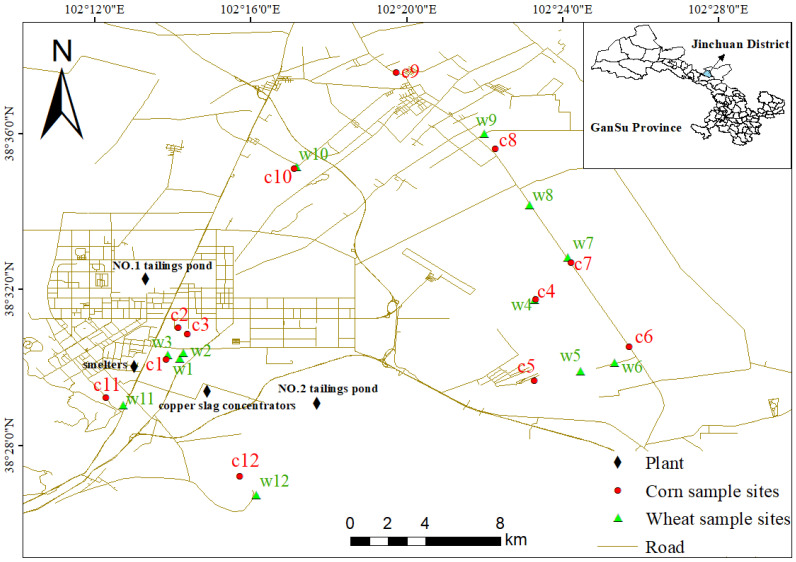
Distribution maps of sampling sites.

The study area was located in Jinchuan District, Jinchang City, Gansu Province, northwestern China (Figure 1), situated in the central Hexi Corridor and characterized by predominant northwest and southeast winds. The soil type was classified as sierozem. Jinchuan District comprises 348,700 mu (23,250 hectares) of urban, rural, and industrial/mining land, with cultivated land spanning 1.84 million mu (122,667 hectares). Crops, including wheat, corn, rapeseed, and legumes, are cultivated across 1.36 million mu (≈90,667 hectares) of sown area, of which wheat dominates, accounting for over 60% of total cultivation. The region hosts globally significant nickel–copper sulfide deposits, ranking third in reserves worldwide and first in China. Industrial activity began in the 1960s with the commissioning of China’s first nickel smelting line. Today, the area contains one metallurgical plant, one copper slag concentrator facility, and two tailings ponds. Prolonged resource exploitation has reduced the region’s environmental carrying capacity, compounded by agricultural practices such as fertilizer, pesticide, and livestock manure application. Consequently, soils near smelting facilities exhibit varying degrees of heavy metal contamination, with metallurgical dust and slag heaps identified as the most severe pollution sources [22].

### 2.2. Sample Collection and Pre-Treatment

Field sampling was conducted in 2022 prior to harvest in farmland soils adjacent to metal smelting facilities and along transportation routes in Jinchuan District (38°26′4″–38°38′16″ N, 102°11′21″–102°26′25″ E). Surface soil samples (0~15 cm depth) and corresponding wheat/corn plants were collected using a stainless-steel sampler. A total of 24 sampling sites were established, with 12 sites each for wheat (labeled w1–w12) and corn (c1–c12) (Figure 1). Soil and crop samples were transported to the laboratory in polyethylene bags. Soil samples were air-dried at room temperature (30 °C), gently ground, sieved through a 10-mesh sieve, and stored in resealable bags. Wheat and corn plants were dissected into five components: roots, stems, leaves, husks, and grains. Plant tissues were rinsed with deionized water, oven-dried at 70 °C to constant weight, pulverized using a grinder, homogenized into powder, and sieved through a 10-mesh sieve. Processed samples were stored in resealable bags for subsequent analytical testing.

### 2.3. Chemical Analysis of Soils and Grains

Heavy metal concentrations in soil and grain samples were determined by atomic absorption spectroscopy (AAS, ZEEnit 700P, Jena Analytical Instruments, Jena, Germany). Soil samples were digested with a HF-HNO_3_-H_2_O_2_ (2:3:1, *v*/*v*/*v*) mixture, while grain samples were digested with HNO_3_-H_2_O_2_ (10:3, *v*/*v*). Following digestion and dilution, the solutions were analyzed by AAS. Soil pH and electrical conductivity (EC) were measured in a 1:2.5 soil–water suspension. Soil organic matter (OM) was quantified by the potassium dichromate volumetric method, and cation exchange capacity (CEC) was determined by the hexaamminecobalt(III) chloride method. Quality control was ensured by nationally certified reference materials GBW07386 (GSS-30) and GBW10023 (GSB-14), with recovery rates maintained at 95–105%. Triplicate measurements were conducted for all soil and crop tissue samples, and results were averaged.

### 2.4. Soil Heavy Metal Pollution Indices

#### 2.4.1. Geo-Accumulation Index

The geo-accumulation index method intuitively evaluates the degree of heavy metal pollution, focusing on the impact of geological factors related to heavy metals and exogenous heavy metals generated by human activities [23,24]. The calculation formula is as follows:

(1) $$I_{\mathrm{geo}}=[C_{i}/(K\cdot B_{i})]$$

where *Igeo* represents the geo-accumulation index for heavy metal i; Ci is the measured concentration of heavy metal i, mg kg^−1^; Bi denotes the local geochemical background value of heavy metal i in the study area, mg kg^−1^; K is the correction coefficient, set to 1.5. Based on *Igeo* values, soil heavy metal pollution levels are classified into six grades: no pollution, mild pollution, moderate pollution, moderately heavy pollution, severe pollution, and extreme pollution, and the corresponding ranges of *Igeo* values are: *Igeo* < 0, 0 ≤ *Igeo* < 1, 1 ≤ *Igeo* < 2, 2 ≤ *Igeo* < 3, 3 ≤ *Igeo* < 4, *Igeo* ≥ 4.

#### 2.4.2. Potential Ecological Risk Index

The potential ecological risk index (RI) evaluates the ecological risks posed by heavy metals in soils by integrating their ecological effects, toxicity, and environmental impacts [25,26]. The methodology is defined as follows:

(2) $$RI=\sum_{i=1}^{n}E_{r}^{i}=T_{i}\times C_{i}\times B_{i}$$

where RI is the comprehensive potential ecological risk index, *Eri* is the potential ecological risk index for heavy metal i, Ti is the toxicity response coefficient of heavy metal i (assigned as Ti = 5 for Cu, Ni, and Co), and Ci is the measured concentration of heavy metal i, mg kg^−1^. Bi denotes the local geochemical background value of heavy metal i in the study area, mg kg^−1^. RI is judged as follows: low ecological risk (RI ≤ 120), moderate ecological risk (120 < RI ≤ 240), and considerable ecological risk (240 < RI ≤ 480). *Eri* is judged as: low ecological risk (*Eri* ≤ 40), moderate ecological risk (40 < *Eri* ≤ 80), and considerable ecological risk (80 < *Eri* ≤ 160).

### 2.5. Bio-Concentration Factor

The bioconcentration factor (BCF), a dimensionless metric, quantifies the transfer of heavy metals from soil to crop tissues and is calculated as follows:

(3) $$BCF=\frac{C_{w}}{C_{s}}$$

where Cw (mg kg^−1^) is the heavy metal concentration in crop tissues; Cs (mg kg^−1^) is the heavy metal concentration in the rhizospheric soil.

### 2.6. Health Risk Assessment

Adopting deterministic risk assessment methods. The non-carcinogenic health risks associated with grain consumption were assessed using the health risk evaluation model established by the US Environmental Protection Agency (USEPA), quantified via the hazard index (*HI*) [27].

The long-term chronic daily intake of heavy metals was calculated as:

(4) $$CDI_{i}=\frac{C_{i}\times IR\times EF\times ED}{BW\times AT}$$

where *CDI* is the chronic daily intake of heavy metals, mg kg^−1^d^−1^; Ci is the measured concentration of heavy metal i, mg kg^−1^; *IR* is the grain ingestion rate, which was 0.412 kg day^−1^ and 0.206 kg day^−1^ for adults and children, respectively, for wheat; and 0.200 kg day^−1^ and 0.100 kg day^−1^ for adults and children, respectively, for corn, determined according to the China Statistical Yearbook 2022; *EF* is exposure frequency, 365 days year^−1^; *ED* is exposure duration: 34 years for adults and 6 years for children; *BW* is the average body weight: 69.6 kg for adults and 15.0 kg for children, respectively; and *AT* stands for averaging time for exposure, with values of *ED* × 365 days year^−1^.

The non-carcinogenic hazard index (*HI*) is calculated as follows:

(5) $$HI=\sum_{i=1}^{n}HQ_{i}=\sum_{i=1}^{n}\frac{CDI_{i}}{RfD_{i}}$$

where *HI* is the non-carcinogenic hazard index, dimensionless; *HQi* is the non-carcinogenic hazard quotient for heavy metal i, dimensionless; *RfDi* is the reference dose for heavy metal I and the *RfD* values of Ni, Cu, and Co are 0.02, 0.04, and 0.0003 mg kg^−1^day^−1^, respectively. These values correspond to the soluble ionic forms. Typically, when *HI* ≤ 1, it indicates negligible non-carcinogenic health risks to humans, while when 1 < *HI* ≤ 10, it suggests potential non-carcinogenic health risks to humans.

### 2.7. Statistical Analysis

Statistical analyses of soil and plant chemical data were conducted using Origin 2022, SPSS 27.0.1, and Microsoft Excel 2021. Sampling site distribution maps and spatial distribution patterns of heavy metals were generated with ArcGIS 10.8.

## 3. Results and Discussion

### 3.1. Soil Physicochemical Properties and Heavy Metal Concentrations

Descriptive statistics of soil properties and heavy metal concentrations in the study area are summarized in Table 1. For wheat-cultivated soils, Ni, Cu, and Co concentrations ranged from 31.65~367.40, 21.50~336.00, and 15.52~36.64 mg kg^−1^, respectively, with mean values of 126.69, 118.41, and 23.61 mg kg^−1^. Coefficients of variation (CV) for these metals followed the order Cu (96%) > Ni (91%) > Co (35%), where the high CVs for Cu and Ni indicate pronounced influence of local mining and metal smelting activities on soil Cu and Ni concentrations [28]. In corn-cultivated soils, Ni, Cu, and Co concentrations spanned 25.43~916.10, 24.10~682.00, and 13.70~50.38 mg kg^−1^, respectively, with mean concentrations of 160.63, 141.58, and 24.47 mg kg^−1^. The CV values exhibited a distinct order: Ni (152%) > Cu (134%) > Co (40%). Notably, Ni and Cu displayed exceptionally high variability (CV > 130%), reflecting extreme site-specific differences in their concentrations, likely attributable to anthropogenic inputs from industrial operations [29].

Compared to regional background values (Table 1), the mean concentrations of Ni, Cu, and Co in Jinchuan District significantly exceeded the soil background levels of Gansu Province. In wheat-cultivated soils, mean Ni, Cu, and Co concentrations were 3.60-, 4.91-, and 1.95-fold higher than local background values, respectively. For corn-cultivated soils, these ratios increased, respectively, to 4.56-, 5.87-, and 2.02-fold; this indicates that local soils have been contaminated to varying degrees. Furthermore, the mean heavy metal concentrations in this study surpassed those reported in industrially impacted regions such as Sargodha, eastern Pakistan [14], and Guilin City, Guangxi Province, China [30]. Notably, Ni concentrations at sampling sites w8, c6, c7, c8, and c10 were lower than the regional background value, likely due to their greater distance from smelting facilities and reduced industrial influence. According to Table 1, 29.17% of sampling sites exceeded China’s risk screening values for Ni, while 37.50% surpassed the Cu threshold, suggesting potential risks to agricultural product safety. These anomalies are attributable to decades of intensive mining and metallurgical activities in the region. Previous work by Batool et al. [14] reported that in a suburban agricultural town of eastern Pakistan, prolonged wastewater irrigation has resulted in cadmium (Cd) accumulation in locally grown wheat exceeding safety thresholds, posing potential health risks to consumers. Studies by Lv et al. [17] identified metal smelting activities as the primary cause of heavy metal contamination in soils surrounding manganese mining areas in Hunan Province, China. Sierozem soils, characterized by organic matter and calcium carbonate content, can immobilize toxic heavy metals through adsorption, limiting their environmental mobility [31]. For instance, in karst soils of southwestern China, Cu predominantly binds to organic matter and carbonate phases, whereas Ni is primarily sequestered in goethite mineral matrices [32,33].

Soil analysis (Table 1) revealed weakly alkaline conditions in the study area, with pH ranging from 7.83 to 8.23. Low electrical conductivity (EC) and organic matter (OM) levels aligned with typical sierozem characteristics. The cation exchange capacity (CEC), reflecting soil nutrient retention potential, ranged from 0.12 to 12.11 cmol^+^ kg^−1^ (mean: 4.46 cmol^+^ kg^−1^), indicating moderate fertility retention capacity. Heavy metal enrichment in soils is influenced by multiple factors, including geogenic background, pedogenic processes, and anthropogenic activities [34,35]. Correlation analyses (Table 2) were conducted to explore relationships among Ni, Cu, Co, pH, EC, OM, and CEC. Pearson correlation coefficients (Table 2) showed significant positive correlations (*p* < 0.01) among Ni, Cu, and Co in agricultural soils, suggesting shared anthropogenic origins and potential synergistic contamination effects. In wheat-cultivated soils, Ni, Cu, and Co exhibited positive correlations with OM (*p* < 0.05), indicating co-accumulation with organic matter. Conversely, no such trend was observed in corn soils. These divergent patterns align with findings by Lasota et al. [6], who reported that correlations between heavy metals and OM may vary across soil datasets. Humic substances in Silesia, Poland, can stabilize heavy metals via metal–organic ligand complexation, potentially explaining the OM-dependent accumulation in wheat soils [6]. Ni and Co in wheat soils showed strong positive correlations with CEC (*p* < 0.01), while Cu correlated moderately (*p* < 0.05). This phenomenon is likely attributable to the greater abundance of negatively charged sites on soil particles at higher CEC levels, which strengthens their adsorption capacity for heavy metals [36]. In corn soils, EC and pH displayed a significant negative correlation (*p* < 0.01), likely driven by reduced soluble salt content in high-pH soils, which lowers ionic conductivity [37].

### 3.2. Spatial Distribution of Heavy Metals in Soils and Crop Grains

**Figure 2:**
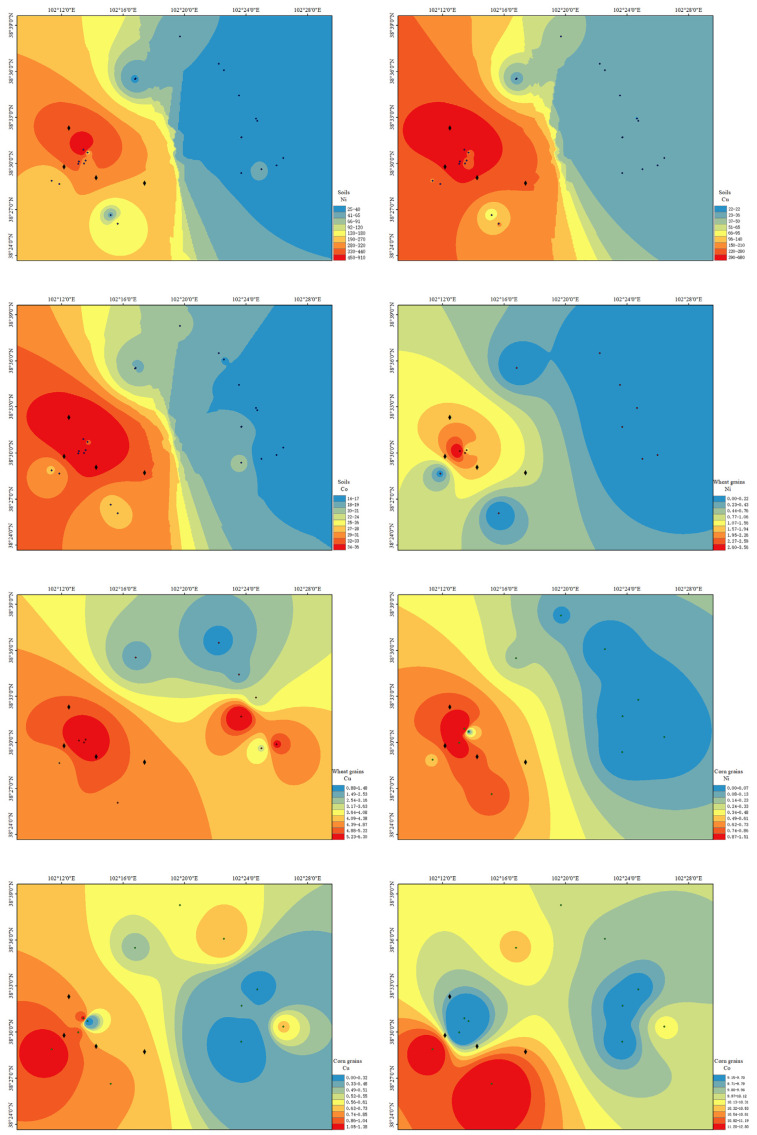
Spatial distribution maps of heavy metals in soils and grains.

Compared to other spatial interpolation methods, the inverse distance weighting (IDW) method more accurately predicts maximum and minimum values and provides better boundary details [38]. Figure 2 displays the spatial distribution maps of heavy metals in soils and crop grains generated using the IDW method. Co in wheat grains was not analyzed due to undetectable levels. Figure 2 shows that the spatial distribution trends of Ni, Cu, and Co in soils were similar, indicating shared sources of heavy metals, consistent with the correlation analysis results. The most severely contaminated soil sampling sites were located near the smelting plant. Based on the prevailing wind direction, the severe heavy metal contamination in soils northwest and southeast of the mining area was likely caused by dust deposition from tailings ponds under wind transport. Other sampling sites, farther from mining activities, showed lesser impacts. In agricultural soils of Anxin County, Hebei Province, the primary sources of Cd, Pb, Zn, and Cu contamination are industrial activities and mobile sources, demonstrating that heavy metals disperse from sources to surrounding areas under human influence [35]. Studies by Anaman et al. [39]. and Xu et al. [40]. highlighted transportation and atmospheric deposition as major factors affecting heavy metal distribution. The spatial distribution results confirm that local metal smelting activities dominate heavy metal contamination.

The spatial distribution of heavy metals in crop grains revealed that Ni distribution in grains closely resembled that in soils, while Cu and Co distributions differed significantly, suggesting soil Ni concentrations can predict grain Ni levels. For wheat grains, Cu hotspots were concentrated near the smelting plant and extended eastward, whereas corn grains showed Cu hotspots near the smelting plant and northeastward, with Co hotspots located south of the plant. The heavy metal content in wheat and corn grains is influenced by soil physicochemical properties, metal speciation, soil microbes and minerals, and crop root exudates [41], which likely explain the spatial discrepancies in Cu and Co distributions between grains and soils.

To further elucidate factors influencing heavy metal translocation from soils to wheat and corn grains, a correlation analysis was conducted between heavy metal concentrations in grains, soils, and soil properties (Table 3). First, Ni and Cu in corn grains showed a significant positive correlation (*p* < 0.01), suggesting shared origins. Notably, while Ni is toxic, Cu is an essential micronutrient for corn, implying potential overlap in their uptake or transport regulatory pathways [42]. This contrasts with findings by Pekel et al. [43], who reported no Ni-Cu correlation in corn grains, possibly due to varietal differences in metal absorption [10,44]. Second, Ni in grains correlated strongly with soil Ni, Cu, and Co (*p* < 0.01), indicating that elevated soil metal levels enhance Ni accumulation in grains, consistent with spatial distribution predictions. Conversely, grain Cu and Co showed no significant correlations with soil metals (Ni, Cu, Co), aligning with results from Wu et al. [45], which may reflect sub-threshold soil metal concentrations for phytotoxicity. According to Deng et al. [46], Ni and Co undergo continuous translocation between phloem and xylem, ultimately redistributing via phloem to plant tissues. Third, Ni in wheat grains correlated significantly with soil OM (*p* < 0.05) and CEC (*p* < 0.01), suggesting that organic chelators in soil enhance Ni bioavailability and plant uptake [47]. While Cu in grains and Co in corn grains showed no significant links to soil properties, the relationship between grain metals and bioavailable soil fractions warrants further exploration.

### 3.3. BCFs of Ni, Cu and Co

Table 4 presents the mean bioconcentration factor (BCF) values across different tissues of wheat and maize plants. For wheat plants, the average BCFs of Ni, Cu, and Co followed the order: leaves > roots > husks > stems > grains, roots > leaves > grains > husks > stems and roots > leaves > stems > husks > grains, respectively. In corn plants, the orders were, respectively, leaves > roots > stems > husks > grains (Ni), leaves > roots > stems > husks > grains (Cu) and grains > husks > leaves > stems > roots (Co). BCF values indicated higher accumulation of Ni and Cu in roots and leaves compared to other tissues. Podar and Maathuis [48] reported that cereals inherently restrict heavy metal translocation from roots to edible aerial parts. Liu et al. [49] demonstrated that atmospheric heavy metals can infiltrate plants via foliar uptake. In this study, elevated Co levels in corn grains may stem from atmospheric dust deposition, with taller corn plants being more susceptible to airborne contamination. Studies by Ma et al. [50] and Xu et al. [19] consistently demonstrate that plant roots serve as the primary site of heavy metal accumulation. This may be attributed to the immobilization of heavy metals through chelation by root cell walls, which restricts their entry into the cytoplasm, or the presence of the Casparian strip that further impedes metal translocation [41].

As shown in Table 4, the BCFs of Ni, Cu, and Co in wheat roots were 1.46-, 2.24-, and 5.77-fold higher than those in corn roots, respectively, indicating that wheat is more susceptible to heavy metal contamination. Similar conclusions were reported by Wu et al. [16] and Xue et al. [51] in agricultural regions of Henan and Hebei Provinces, China, suggesting that wheat exhibits greater sensitivity to heavy metal stress than corn, posing higher health risks to humans. Rezapour et al. [52] demonstrated that wheat exhibits stronger translocation of heavy metals from soil to roots than from roots to grains. In contrast, Romdhane et al. [53] found that corn preferentially transfers heavy metals to stems and leaves. To assess the safety of locally produced crop straw as livestock feed, mean heavy metal concentrations in wheat and corn tissues are presented in Table 5. Results indicated that, according to the Safety Guidelines for Feed Additives, Co concentrations in all wheat and corn tissues exceeded regulatory limits except for wheat grains and corn roots/stems. Similarly, Cu concentrations in wheat roots, corn leaves, and wheat leaves surpassed permissible thresholds, confirming that straw from these crops is unsuitable for use as standalone animal feed.

### 3.4. Soil Pollution and Health Risk Assessment

Table 6 summarizes the percentages of sampling sites classified under different geo-accumulation index (*Igeo*) and single-element potential ecological risk index (*Eri*) levels. According to *Igeo* criteria, 21% and 37% of sampling sites for Ni and Cu, respectively, exhibited contamination levels of moderately heavy pollution or higher, with 4% of sites for both metals reaching extreme pollution, indicating significant accumulation of Ni and Cu in regional soils. All Co-contaminated sites were classified as moderate pollution or lower. For *Eri*, 12% (Ni) and 29% (Cu) of sampling sites posed moderate ecological risk or higher, with 4% of sites for both metals categorized as considerable ecological risk, suggesting potential threats to human health via food chain accumulation. Co-associated risks remained low across all sites. Overall, the descending order of contamination and ecological risk levels for all metals was: Cu > Ni > Co.

**Figure 3:**
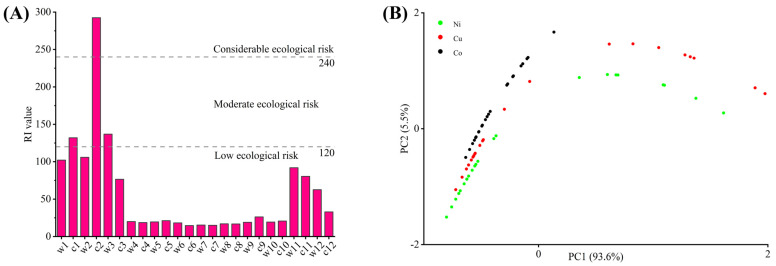
Results of the evaluation of potential ecological risks (**A**). PCA plot of total heavy metal concentration in soil and risks of *Igeo* and *Eri* (**B**).

As shown in Table 6, the three elements in soils were significantly altered by production activities from mining enterprises near farmland, leading to severe spatial heterogeneity in heavy metal distribution. The potential ecological risk (RI) assessment (Figure 3A) revealed the highest ecological risk at sampling site c2, classified as considerable ecological risk, followed by c1 and w3 with moderate ecological risk, all located near local mining areas. Sites adjacent to mining zones (w1, w2, c3, w11, c11, w12, c12) exhibited elevated RI values within the low ecological risk category, further confirming mining activities as the primary contamination source. Elevated risks at other sites likely stem from persistent dust deposition linked to metal smelting and vehicular emissions [4]. Given the irreversible ecological impacts of heavy metal pollution in agricultural soils, immediate remediation measures are urgently required for this region.

To better understand contamination characteristics, *Igeo*, *Eri* and total heavy metal concentrations in soils were incorporated into principal component analysis (PCA). As shown in Figure 3B, two principal components were extracted: PC1, dominated by *Eri* and total metal concentrations, explained 93.6% of the total variance, while PC2, driven by *Igeo* values, accounted for 5.5%. Based on Figure 3B, the *Igeo* levels approximately ranked as Co > Cu > Ni, indicating multi-metal composite pollution with high similarity among the three metals, being consistent with spatial distribution predictions. The PCA results suggest that spatial heterogeneity of heavy metal concentrations is the primary factor influencing contamination patterns. The high explanatory power of PC1 underscores anthropogenic interventions, particularly mining and smelting activities, as key drivers of heavy metal distribution across the study area.

**Figure 4:**
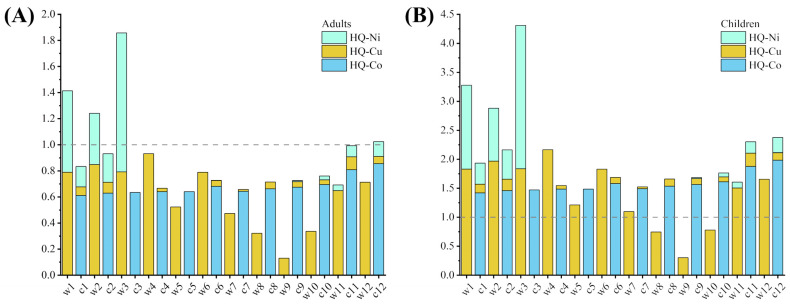
Non-Carcinogenic Health Risk Index (*HI*) for Adults (**A**) and Children (**B**).

Wheat and corn, as staple crops in Jinchuan District, Jinchang City, are closely linked to the health of local residents. Table 7 presents the chronic daily intake (*CDI*) of heavy metals for adults and children. Most studies indicated that children face higher non-carcinogenic risks from heavy metals in grains compared to adults [5,54]. In this study, wheat was identified as the primary source of Ni and Cu exposure, while Co exposure predominantly originated from corn. The *CDI* values for children were 2.32-fold higher than those for adults. The non-carcinogenic hazard index (*HI*) results are shown in Figure 4. *HI* values in this study revealed that children in Jinchuan District face greater non-carcinogenic health risks than adults, with the highest risk observed for wheat from site w3 (*HI* = 4.309), followed by w1 (3.279) and w2 (2.881). Notably, 16.67% of adults and 87.50% of children in the study area were exposed to non-carcinogenic health risks. Approximately 75% of children exhibited hazard quotients (*HQ*) > 1 for Cu in wheat, while all children showed *HQ* > 1 for Co in corn, indicating that Cu in wheat and Co in corn are the primary contributors to *HI* among the three heavy metals. Health risk assessments indicate that children face greater harm, likely due to their lower body weight and higher metabolic rates [55]. Furthermore, long-term heavy metal exposure may cause irreversible impairments to neurological and immune systems in children [11]—an urgent public health priority requiring immediate attention.

For individuals living near metal smelting zones, heavy metals can also enter the human body through inhalation and dermal contact, potentially leading to higher non-carcinogenic health risks [56]. Based on this study, the following recommendations are proposed for local agricultural practices: In areas corresponding to sampling sites c8, c9, and c10, corn cultivation poses health risks exceeding safety thresholds, while wheat cultivation presents lower risks. Therefore, replacing corn with wheat or alternative crops in these regions is advised to reduce heavy metal exposure for residents. Other sampling sites are no longer suitable for growing wheat or corn intended for human consumption, and soil remediation measures should be implemented in these farmlands.

## 4. Conclusions

The mean concentrations of Ni and Cu in the study area exceeded China’s risk screening values for agricultural soils, while Co surpassed the background levels of Gansu Province. Correlation analyses revealed a high homology among Ni, Cu, and Co in soils, indicating shared anthropogenic origins. Spatial distribution patterns identified metal smelting facilities as the primary source of soil contamination. Ni in crop grains closely mirrored its spatial distribution in soils, enabling soil Ni levels to predict grain Ni contamination, whereas Cu and Co exhibited divergent grain-soil distribution trends. The correlation between grains and soils confirmed that soil is the dominant source of heavy metals in crops. Wheat demonstrated greater susceptibility to heavy metal contamination than corn, and straw from both crops was unsuitable for direct use as animal feed. Soil contamination levels followed the descending order: Cu > Ni > Co. Health risk assessments identified wheat as the principal contributor to Ni and Cu exposure, while corn dominated Co exposure. Given the elevated non-carcinogenic risks, cultivating wheat and corn for human consumption in the study area is no longer viable, necessitating immediate soil remediation.

This study not only confirms severe complex heavy metal contamination in the region, but also systematically reveals—for the first time—the shared sources and system characteristics of Ni, Cu, and Co pollution, their crop-specific uptake patterns, and the differential health risks they pose particularly to children. This study provides actionable insights for local agricultural restructuring and establishes a transferable risk management framework for mining-impacted cities globally. A key limitation lies in the use of total heavy metal concentrations for risk assessment. Future efforts should prioritize speciation analysis to determine bioavailability, alongside mechanistic investigations into crop-specific uptake and intra-grain translocation of heavy metal fractions.

## Figures and Tables

**Table 1:** Descriptive statistics of soil properties and heavy metal concentrations in agricultural soils of study area and related soil quality standards (mg kg^−1^).

	Statistical Values	Ni	Cu	Co	pH	EC (μS cm^−1^)	OM (g kg^−1^)	CEC (cmol^+^ kg^−1^)
Wheat soil (n = 12)	Max	367.40	336.00	36.64	8.15	347.00	40.90	12.11
	Min	31.65	21.50	15.52	7.83	170.80	20.67	0.49
	Mean	126.69	118.41	23.61	8.00	253.53	29.90	5.00
	Standard deviation (SD)	115.57	113.23	8.17	0.10	56.75	6.41	3.94
	Coefficient of variation (CV)	0.91	0.96	0.35	0.01	0.22	0.21	0.79
Corn soil (n = 12)	Max	916.10	682.00	50.38	8.23	324.00	38.87	8.97
	Min	25.43	24.10	13.70	7.92	147.00	14.26	0.12
	Mean	160.63	141.58	24.47	8.13	193.72	28.74	3.92
	Standard deviation (SD)	244.83	189.64	9.77	0.08	54.10	6.65	2.62
	Coefficient of variation (CV)	1.52	1.34	0.40	0.01	0.28	0.23	0.67
Reference standard	Soil background values of Gansu Province	35.20	24.10	12.10				
	Risk screening value of agricultural land soil	190.00	100.00	——				

**Table 2:** Pearson correlation coefficients between heavy metals and other parameters in soils.

Soils	Ni	Cu	Co	pH	EC	OM	CEC
Ni	1	0.986 **	0.935 **	−0.172	−0.339	0.240	−0.174
Cu	**0.987** **	1	0.952 **	−0.158	−0.370	0.305	−0.201
Co	**0.972** **	0.970 **	1	−0.233	−0.312	0.183	−0.204
pH	**0.091**	**−0.009**	**−0.048**	1	−0.781 **	0.437	−0.200
EC	**−0.191**	**−0.157**	**−0.174**	**−0.123**	1	−0.414	0.438
OM	**0.646** *	**0.612** *	**0.577** *	**0.156**	**0.260**	1	0.295
CEC	**0.726** **	**0.662** *	**0.722** **	**0.194**	**−0.197**	**0.484**	1

Bold represents the correlation coefficients of wheat, and the other stands for corn. ** *p* < 0.01. * *p* < 0.05.

**Table 3:** Pearson correlation coefficients for metal concentrations in wheat and corn grains, total soil metal contents and soil properties.

Grain	Ni	Cu	Co
Grain			
Ni	1	0.714 **	0.138
Cu	**0.429**	1	0.428
Co	**NA**	**NA**	1
Soil			
Ni	0.887 ** (0.831 **)	**0.540** (0.504)	**NA** (−0.243)
Cu	0.817 ** (0.877 **)	**0.524** (0.559)	**NA** (−0.211)
Co	0.782 ** (0.885 **)	**0.573** (0.499)	**NA** (−0.056)
other			
pH	**0.466** (−0.243)	**0.168** (0.048)	**NA** (−0.059)
EC	**−0.240** (−0.339)	**0.260** (−0.486)	**NA** (−0.090)
OM	0.686 * (0.050)	**0.324** (0.190)	**NA** (−0.352)
CEC	0.728 ** (−0.363)	**0.191** (−0.244)	**NA** (−0.078)

Bold represents the correlation coefficients of wheat, and the other stands for corn. ** *p* < 0.01. * *p* < 0.05. NA: not available.

**Table 4:** Average bio-concentration factor (BCF) of Ni, Cu and Co in different parts of crop plants.

Roots			Stems			Leaves			Husks			Grains		
Ni	Cu	Co	Ni	Cu	Co	Ni	Cu	Co	Ni	Cu	Co	Ni	Cu	Co
**0.164**	**0.215**	**0.381**	**0.011**	**0.061**	**0.149**	**0.251**	**0.204**	**0.258**	**0.078**	**0.071**	**0.119**	**0.002**	**0.079**	**0.000**
0.112	0.096	0.066	0.030	0.090	0.068	0.124	0.300	0.180	0.014	0.058	0.368	0.002	0.010	0.471

Bold represents the BCF of wheat, and the other stands for corn.

**Table 5:** Mean heavy metal concentrations in different parts of wheat and corn (mg kg^−1^).

	Roots			Stems			Leaves			Husks			Grains		
	Ni	Cu	Co	Ni	Cu	Co	Ni	Cu	Co	Ni	Cu	Co	Ni	Cu	Co
Wheat	20.57 ± 21.11	22.93 ± 24.20	8.56 ± 2.41	1.50 ± 1.61	3.77 ± 2.30	3.00 ± 2.18	28.87 ± 25.82	22.88 ± 22.52	6.48 ± 4.16	7.25 ± 5.36	5.56 ± 4.42	2.73 ± 0.98	0.60 ± 1.16	4.11 ± 1.69	0.00 ± 0.00
Corn	13.18 ± 17.24	10.39 ± 11.35	1.75 ± 1.70	2.31 ± 1.81	5.37 ± 3.87	1.53 ± 0.64	16.19 ± 22.02	25.94 ± 26.90	4.44 ± 2.51	3.08 ± 5.53	5.38 ± 5.94	8.05 ± 0.73	0.36 ± 0.52	0.60 ± 0.43	10.20 ± 1.12

The data are expressed as the means ± standard deviations (mean ± SDs) of 12 sampling sites (n = 12). Code of practice for the safe use of feed additives: 15 mg kg^−1^ for Cu and 2 mg kg^−1^ for Co in feed, with no limit for Ni.

**Table 6:** Percentage of sampling points in different *Igeo* and *Eri* risk levels in Jinchuan District.

		Ni (%)	Cu (%)	Co (%)
	Class			
*Igeo*	0	54	54	33
	1	8	8	58
	2	17	0	8
	3	17	25	0
	4	0	8	0
	5	4	4	0
$E_{r}^{i}$	<40	88	71	100
	40~80	8	25	0
	80~160	4	4	0

**Table 7:** *CDI* mean values of wheat and corn grains (mg kg^−1^ d^−1^).

	*CDI* (Ni)		*CDI* (Cu)		*CDI* (Co)	
	Adults	Children	Adults	Children	Adults	Children
Wheat	3.55 × 10^−3^	8.23 × 10^−3^	2.43 × 10^−2^	5.64 × 10^−2^	0.00	0.00
Corn	1.02 × 10^−3^	2.37 × 10^−3^	1.73 × 10^−3^	4.02 × 10^−3^	2.93 × 10^−2^	6.80 × 10^−2^

## Data Availability

The datasets generated for this study are available on request to the corresponding author.

## Abbreviations

*Igeo*	The geo-accumulation index
*RI*	The potential ecological risk index
*BCF*	The bioconcentration factor
*HI*	The hazard index
*CDI*	The chronic daily intake
